# Epidemiological study and risk factors of internal parasite infections in wild boar (*Sus scrofa*) in Mazandaran Province, northern Iran

**DOI:** 10.1016/j.parepi.2026.e00529

**Published:** 2026-08-03

**Authors:** Moein Abolhasani Drounkola, Mohammad Reza Salimi Bejestani, Emad Changizi

**Affiliations:** Department of Pathobiology, Faculty of Veterinary Medicine, Semnan University, Semnan, Iran

**Keywords:** Acanthocephala, Cestoda, Nematoda, Protozoa, Trematoda, Wild boar

## Abstract

**Background/objective:**

Given the importance of zoonotic diseases among wildlife, domestic animals, and humans, and the role of wild animals as natural reservoirs of many pathogens, this study was designed to investigate the epidemiology and identify risk factors associated with internal parasitic infections in wild boar (*Sus scrofa*) in Mazandaran province.

**Methods:**

From January 2021 to December 2023, 15 wild boars, including 6 males and 9 females, that died in Mazandaran Province, northern Iran, were collected, those that were killed in car accidents or illnesses, and transferred to the Veterinary Parasitology Laboratory of Semnan University, Semnan, Iran, for parasite examination. The contents of the gastrointestinal (GI) tract were sieved, and the mucosa was examined for parasites. For the liver, the bile duct was examined, and for the lungs, the bronchi of both lobes were cut off, and all the parasites in this area were found, sieved, and washed, so that all the remaining worms were collected in the sieve. For muscle examination, several methods were used, including the squash prep test, tissue digestion, pathological assay, and Dab smear.

**Results:**

All of the boars had at least one parasite, and 13 species of helminths and protozoa were identified. Eight species of nematodes were identified, including *Ascarops strongylina* (33.3%), *Globocephalus urosubulatus* (26.6%), *Gongylonema pulchrum* (6.6%)*, Metastrongylus apri* (53.3%), *Metastrongylus pudendotectus* (46.6%), *Oesophagostomum dentatum* (20%)*, Physocephalus sexalatus* (33.3%), and *Trichuris suis* (6.6%). Moreover, two species of trematodes (*Dicricoelium dendriticum* (13.3%) and *Fasciola hepatica* (6.6%)), one species of Acanthocephala (*Macracanthorhynchus hirudinaceus* (86.6%)), and one protozoan (*Sarcocyst* spp. (26.6%)) were identified*.* In addition, larvae of *Taenia hydatigena* were observed*.*

**Conclusion:**

Wild boar populations in Mazandaran province exhibit a high prevalence of internal parasitic infections, indicating the active presence of parasitic cycles in the region's natural habitats.

## 1 Introduction

Wild boar (*Sus scrofa*), one of the most widespread ungulate species in the world, plays a significant ecological role across various ecosystems (Barrios-Garcia and Ballari, 2012). Due to its high adaptability and high reproductive rate, this species can live in a wide range of habitats, from dense forests to agricultural areas to the outskirts of cities (Markov et al., 2022; Rekiel et al., 2024; Rivero et al., 2019). This behavioral and nutritional flexibility has increased interactions among these animals, domestic animals, and humans. Meanwhile, boar populations are known to be important reservoirs and potential carriers of a variety of zoonotic pathogens, especially internal parasites, which underscores the importance of studying their health in the context of public health and veterinary medicine (Fredriksson-Ahomaa, 2019; Jota Baptista et al., 2023; Maleki et al., 2020).

In Iran, wild boars are found primarily in marshes, oak forests across the Alborz and Zagros mountains, and regions with high vegetation density (Maleki et al., 2020). They can also be found in southern Iran, in areas such as the Arjan plain, where they graze under wild pistachio trees, and in northern Iran, in the mountains and the Caspian Hyrcanian mixed forests (Ashrafzadeh et al., 2018; Khalilzadeh et al., 2016).

Internal parasitic infections can have profound effects on the health and population dynamics of wild boars, ranging from reduced fitness and physical weakness to increased susceptibility to other diseases and, in severe cases, mortality (Meng et al., 2009; Perin et al., 2023; Petersen et al., 2020; Ruiz-Fons et al., 2008). From an economic perspective, internal parasitic infections can also cause significant damage to the livestock industry by being transmitted to domestic animals, such as pigs and cattle (Brewer et al., 2025; Gorji et al., 2026; Lopes et al., 2016).

Zoonoses caused by parasites of wild animals, especially wild boar, are a major public health challenge in many regions. Wild boars act as natural reservoirs for a wide range of parasitic agents with zoonotic potential, including *Trichinella* spp., *Toxoplasma gondii*, *Taenia* spp., and *Fasciola* spp., which can be transmitted to humans through the consumption of raw or undercooked meat (Jota Baptista et al., 2023; Maleki et al., 2020; Odeniran and Ademola, 2016). Despite the extensive emphasis on zoonotic aspects in previous studies, many studies in the study area have not directly identified these species, and references to parasites such as *Trichinella* have often been made without support from local epidemiological data.

Given the importance of zoonotic diseases among wildlife, domestic animals, and humans, and the role of wild animals as natural reservoirs of many pathogens, this study was designed to investigate the epidemiology and identify risk factors associated with internal parasitic infections in wild boar (*Sus scrofa*) in Mazandaran province. The primary focus of this study is to determine the prevalence, infection severity, and parasite species diversity.

## 2 Materials and methods

### 2.1 Geographical study area

Mazandaran Province is located in northern Iran, on the southern coast of the Caspian Sea (Zaker et al., 2007). Geographically, the region is bordered by the Alborz Mountains to the south and the Caspian Sea to the north, and has a temperate, humid Caspian climate that distinguishes it from Iran's central plateau. The special geographical location of Mazandaran, the presence of dense Hyrcanian forests, fertile agricultural lands, and a dense hydrographic network comprising numerous rivers originating in the Alborz and flowing into the Caspian Sea, has led to the formation of diverse natural landscapes and unique ecosystems (Hosseini et al., 2025; Tohidifar et al., 2016). These features have made Mazandaran Province an attractive and important study area for geographical research.

### 2.2 Study design

From January 2021 to December 2023, 15 wild boars, including 6 males and 9 females, that died in Mazandaran Province, northern Iran, were collected, those that were killed in car accidents or illnesses, and transferred to the Veterinary Parasitology Laboratory of Semnan University, Semnan, Iran, for parasite examination.

### 2.3 Necropsy examination and parasite detection

Investigated organs included the lung, liver, kidney, muscles, and the gastrointestinal tract, including the mouth and esophagus. All organs were first visually examined for parasites and lesions. The contents of the GI tract were sieved, and the mucosa was examined for parasites. For the liver, the bile duct was examined, and for the lungs, the bronchi of both lobes were cut off, and all the parasites in this area were found, sieved, and washed, so that all the remaining worms were collected in the sieve. For muscle examination, several methods were used, including the squash prep test, tissue digestion, pathological assay, and Dab smear. After collection, the helminths were placed in lactophenol (Ahmadi et al., 2025), Acanthocephalans and Trematodes stained with carmine, and identified by morphology (Boch, 2006; Gibbons, 2010; Moravec, 2009; Morita et al., 2007; Schmidt, 1972; Soulsby, 1982).

## 3 Results

**Fig. 1:**
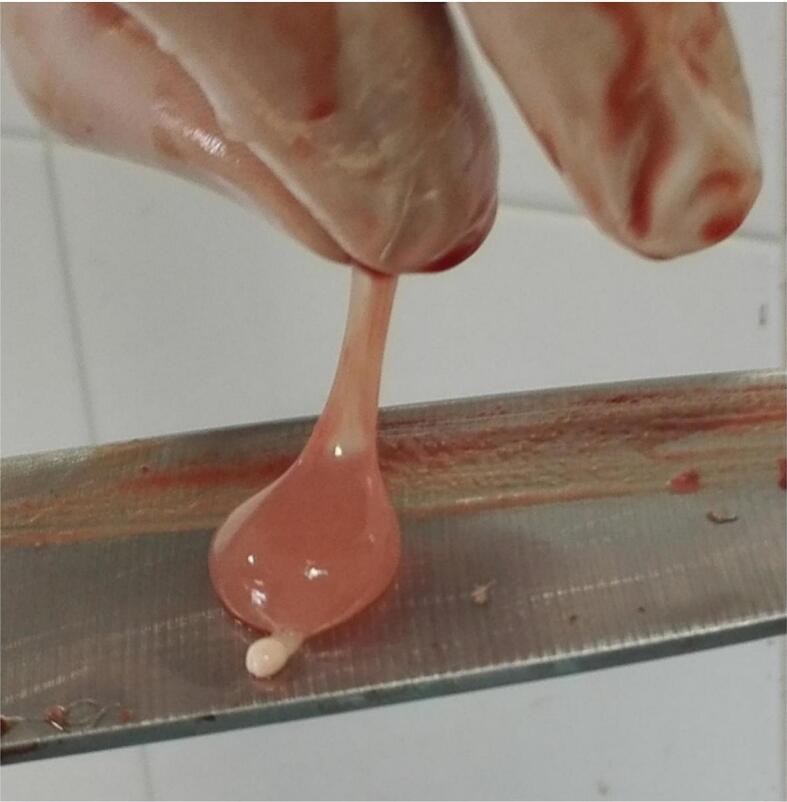
*Taenia hydatigena* larvae (Cysticercus tenuicollis).

In total, thirteen species of helminths and protozoa were identified. Eight species of nematodes were identified, including *A. strongylina* (33.3%), *G. urosubulatus* (26.6%), *G. pulchrum* (6.6%)*, M. apri* (53.3%), *M. pudendotectus* (46.6%), *O. dentatum* (20%)*, P. sexalatus* (33.3%), and *T. suis* (6.6%). Moreover, two species of trematodes (*D. dendriticum* (13.3%) and *F. hepatica* (6.6%)), one species of Acanthocephala (*M. hirudinaceus* (86.6%)), and one protozoan (*Sarcocystis* spp. (26.6%)) were identified*.* In addition, *T. hydatigena* larvae were observed (Fig. 1)*.*

**Fig. 2:**
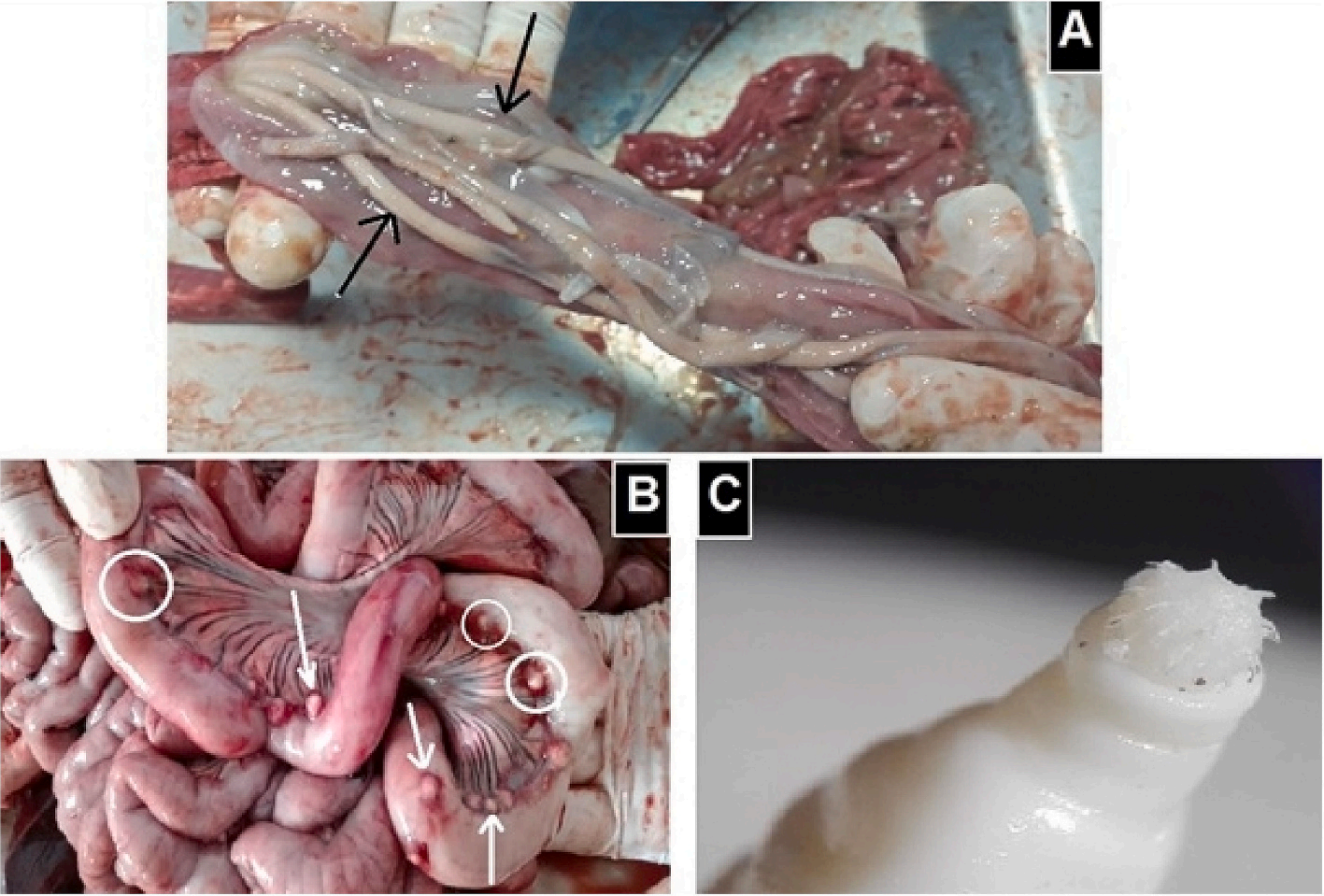
(A) *Macracanthorhynchus hirudinaceus* in the small intestine. (B) Nodules on the small intestine due to these parasites. (C) Anterior end, showing the hooks.

**Fig. 3:**
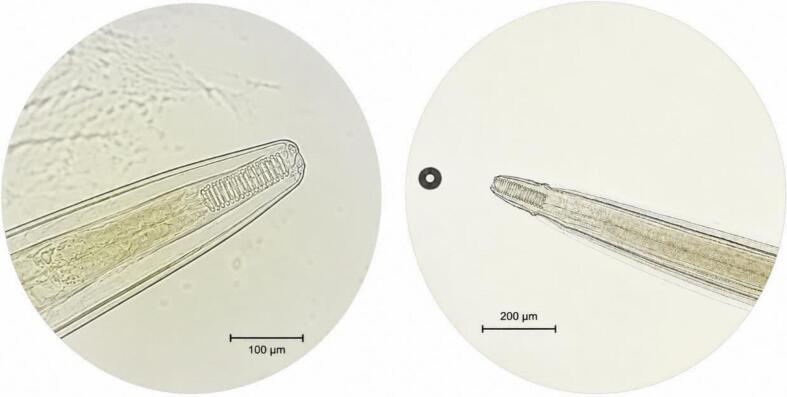
(A) Lateral view of the anterior end of *Ascarops strongylina.* (B) Lateral view of the anterior end of *Physocephalus sexalatus.*

**Fig. 4:**
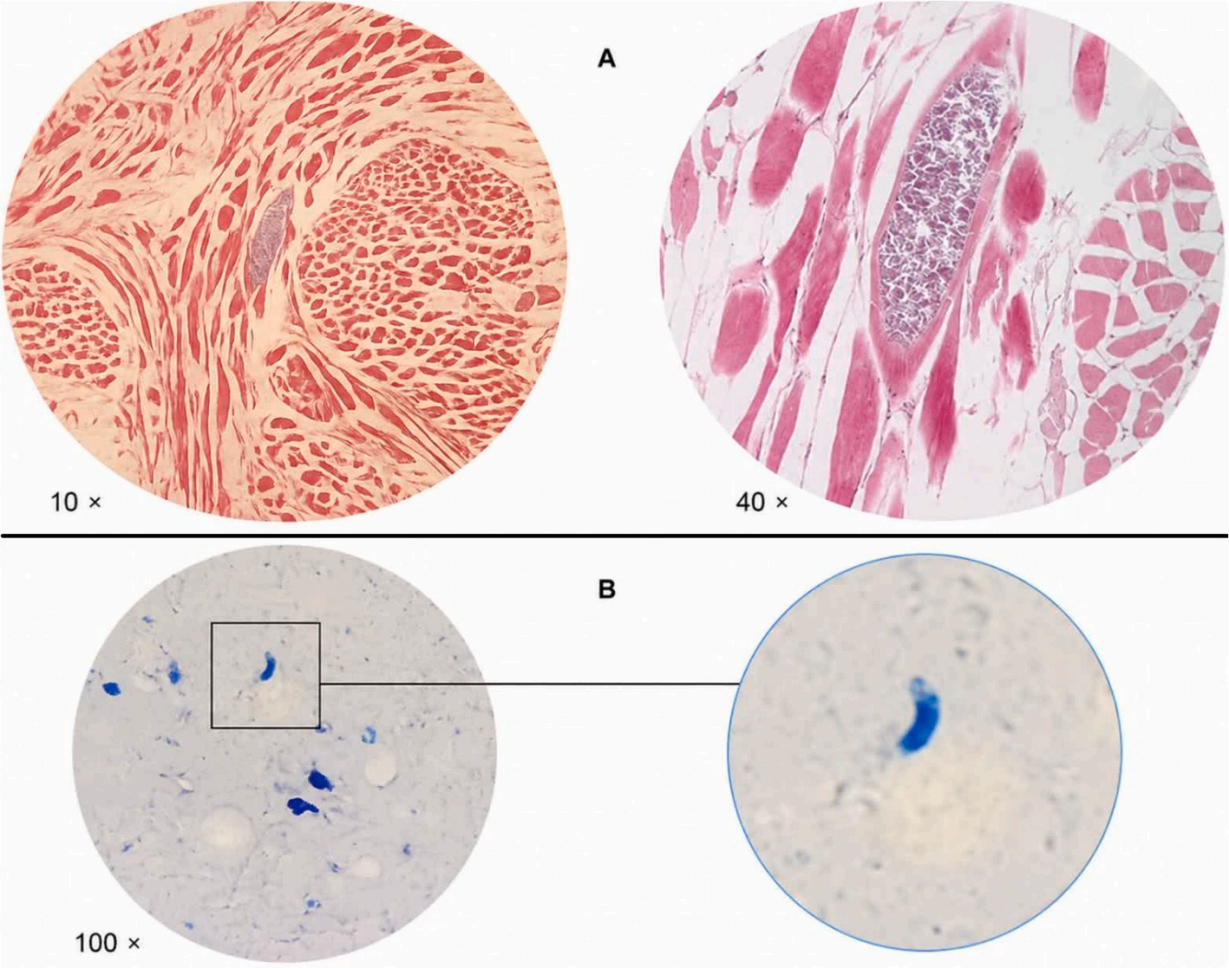
(A) *Sarcocyst* spp. cyst in the wild boar tongue muscle. (B) *Sarcocyst* bradyzoite*.*

**Fig. 5:**
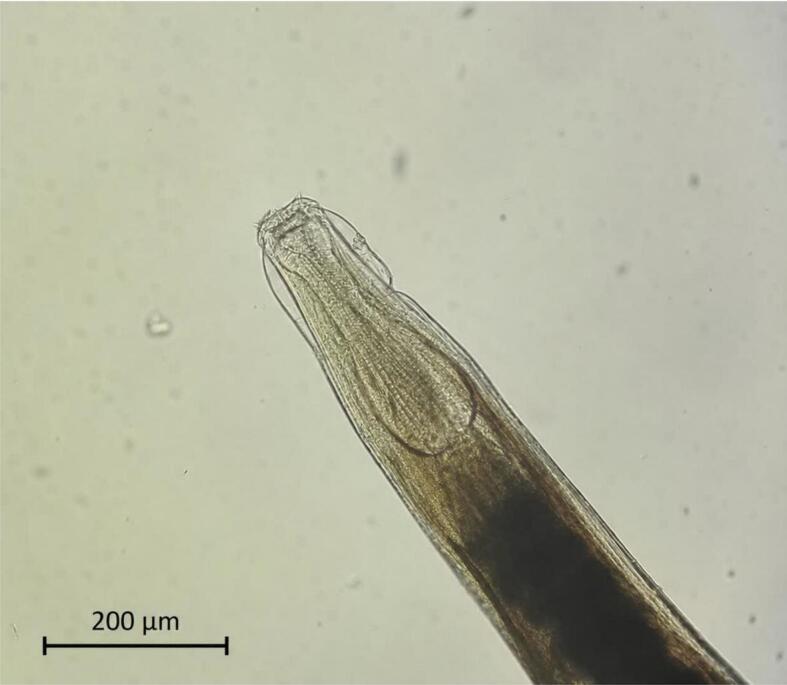
Lateral view of the anterior end of *Oesophagostomum dentatum.*

Among these parasites*,* the *M. hirudinaceus* (Fig. 2) was found in all 13 wild boars. Next, *M. apri* from 8*, M. pudendotectus* from 7*, A. strongylina* (Fig. 3), and *P. sexalatus* from 5, *G. urosubulatus,* and *Sarcosyst* spp. (Fig. 4) from 4, *O. dentatum* (Fig. 5) from 3, and *D. dendriticum* from 2 wild boar were identified*.* Other parasites were only extracted from one wild boar (Table 1).

**Table 1:** Prevalence, mean intensity, and range of helminth species in 15 wild boars.

Organ	Parasite	Prevalence no. (%)	Mean Intensity (mean ± SD)	Range	Total no.
Liver	*Dicrocoelium dendriticum*	2 (13.3)	17.50 ± 4.95	14–21	35
	*Fasciola hepatica*	1 (6.6)	1 ± 0	1	1
Lung	*Metastrongylus apri*	8 (53.3)	24.88 ± 32.51	5–104	199
	*Metastrongylus pudendotectus*	7 (46.6)	13.71 ± 9.05	4–29	96
Esophagus	*Gongylonema pulchrum*	1 (6.6)	2.00 ± 0	2	2
Stomach	*Ascarops strongylina*	5 (33.3)	25.60 ± 25.66	4–70	128
	*Physocephalus sexalatus*	5 (33.3)	8.60 ± 4.98	2–16	43
Small intestine	*Globocephalus urosubulatus*	4 (26.6)	27.75 ± 22.55	11–61	111
	*Macracanthorhynchus hirudinaceus*	13 (86.6)	19.23 ± 17.10	1–61	250
Large intestine	*Trichuris suis*	1 (6.6)	87.00 ± 0	87	87
	*Oesophagostomum dentatum*	3 (20)	16.67 ± 8.08	8–24	50
Mesenteries	*Taenia hydatigena* larvae	1 (6.6)	2.00 ± 0	2	2
Muscle	*Sarcosyst* spp.	4 (26.6)	–	Not counted	–

**Fig. 6:**
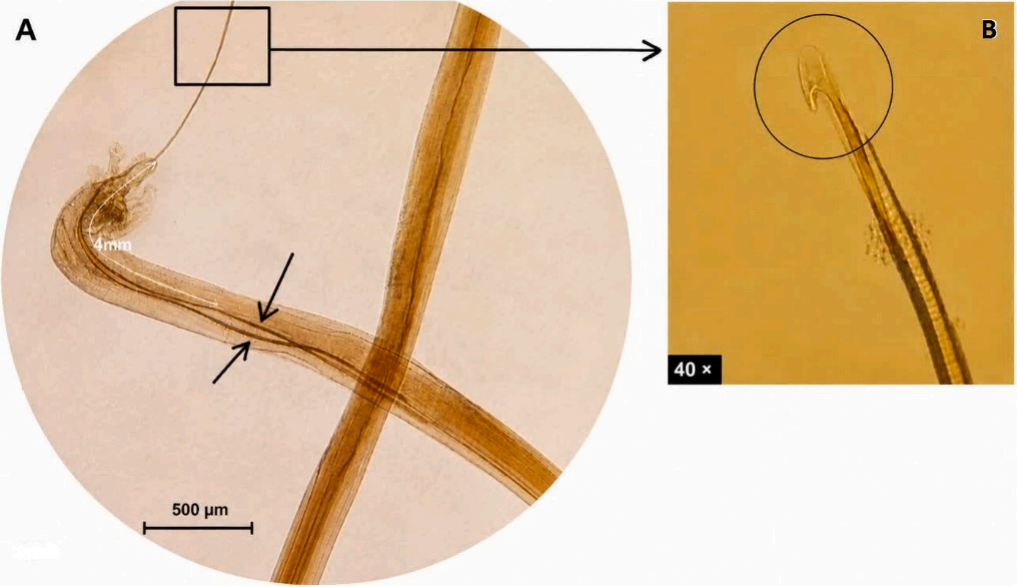
*Metastrongylus apri,* (A) Lateral view of the posterior end of a male, showing the two spicules and their size. (B) End of the spicules.

**Fig. 7:**
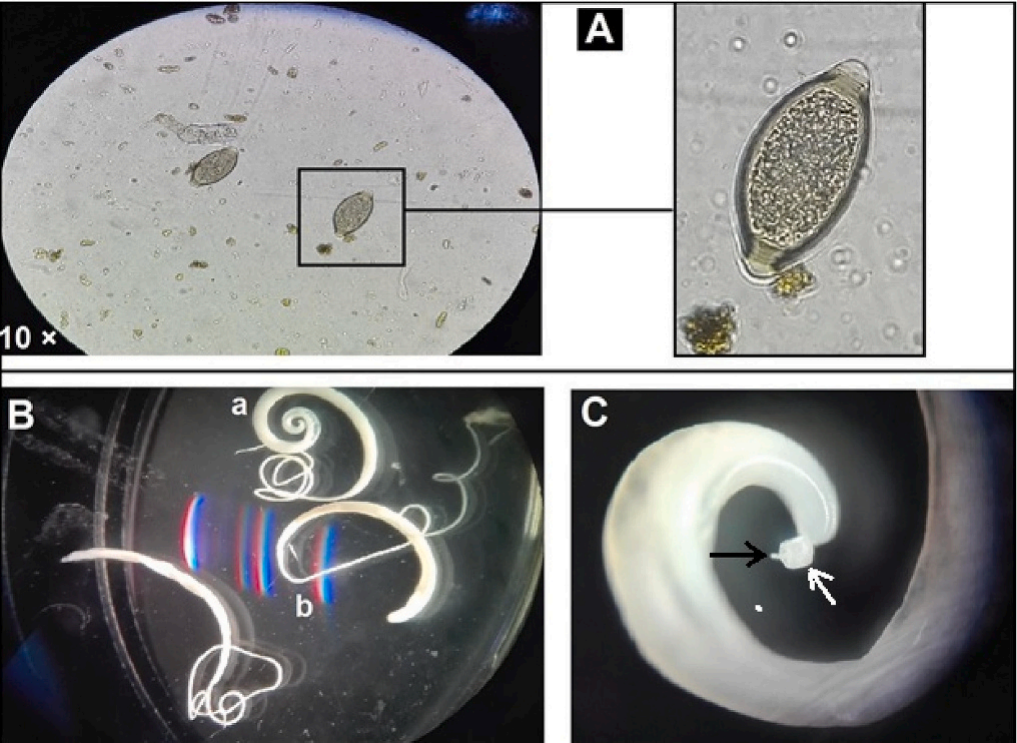
(A) Egg of *Trichuris suis* (B) (a) Whole body view of a male, (b) Whole body view of a female. (C) The posterior end of a male, the spicule, and the spicule sheath are indicated by the arrow.

**Fig. 8:**
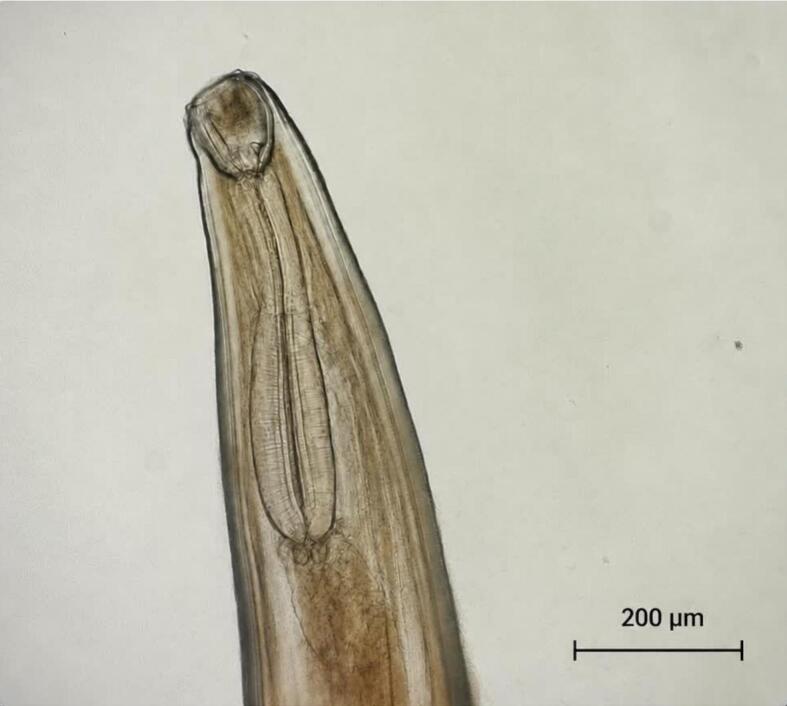
Lateral view of the anterior end of *Globocephalus urosubulatus.*

**Fig. 9:**
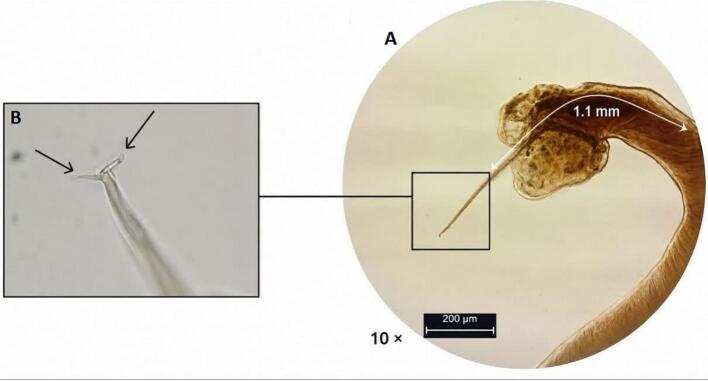
*Metastrongylus pudendotectus,* (A) Lateral view of the posterior end of a male, showing the two spicules and their size. (B) End of the spicules.

**Fig. 10:**
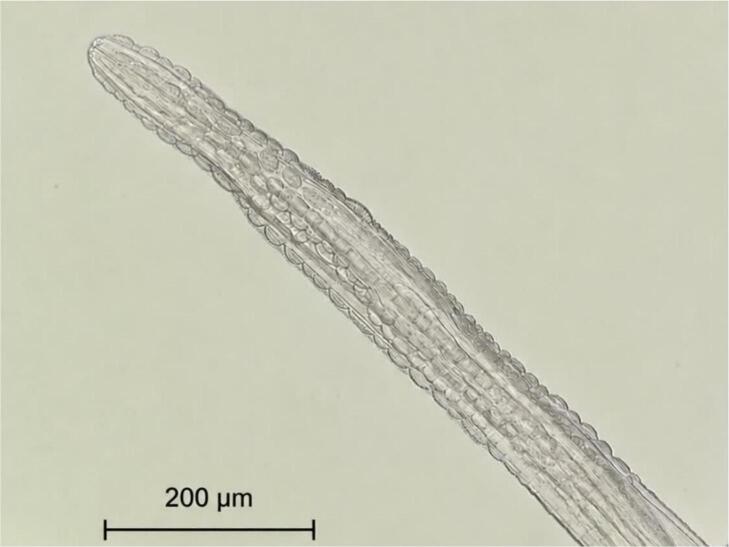
Lateral view of the anterior end of *Gongylonema pulchrum.*

**Fig. 11:**
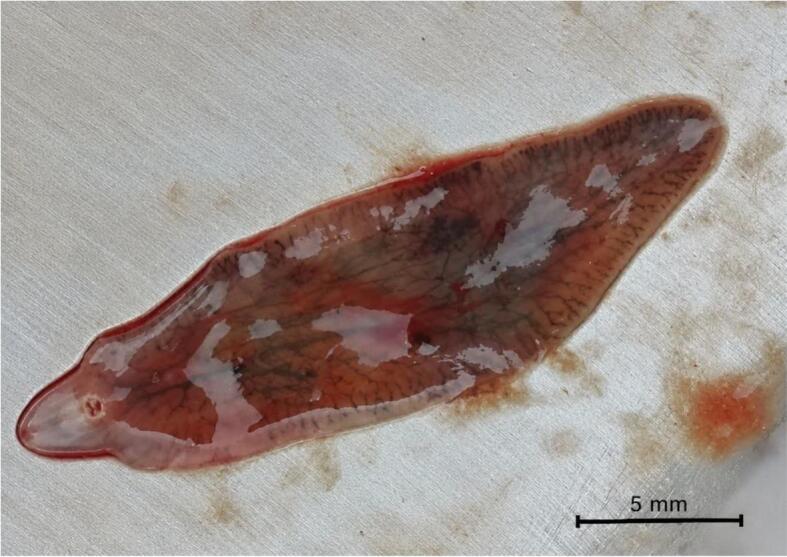
*Fasciola hepatica*.

Regarding the number and frequency of parasites in relevant organs, *M. apri* (Fig. 6), with more than 100 in the lung, has the highest frequency of occurrence among parasites. *T. suis* (Fig. 7) 87, *A. strongylina, G. urosubulatus* (Fig. 8), *M. hirudinaceus*, *M. pudendotectus* (Fig. 9), *O. dentatum*, *D. dendriticum*, *P. sexalatus*, *G. pulchrum* (Fig. 10), *F. hepatica* (Fig. 11), and *Rhipicephalus* spp. have the highest frequency in various organs, respectively.

## 4 Discussion

In the current investigation, 13 out of 15 wild boars were infected with *M. hirudinaceus*, accounting for 86.6% of infections. Based on a previous study in Boushehr province, 13 out of 25 wild boars (52%) were infected by *M. hirudinaceus* (Mansouri et al., 2016), and 64% in Khouzestan province, Iran (Mowlavi et al., 2006). Although Eslami (1992) reported that the number of *M. hirudinaceus* in each infected boar was fewer than 30 (Eslami and Farsad-Hamdi, 1992), one of the necropsied wild boars in this study had 61 *M. hirudinaceus*. Moreover, the prevalence of *Gl. urosubulatus* was 26.6%, while other studies reported 33.3% (Dodangeh et al., 2018), 74% (Eslami and Farsad-Hamdi, 1992), and 22% (Senlik et al., 2011). Our results revealed the highest *M. hirudinaceus* infection rate in wild boars (86.6%) compared with other studies*.*

Only one wild boar was infected with *T. suis,* and the infection intensity was relatively high (61 worms). Previous studies have reported *T. suis* infection rates in wild boars ranging from 7% to 19.4% (Dodangeh et al., 2018; Senlik et al., 2011; Solaymani-Mohammadi et al., 2003). Moreover, histological examinations in the current study showed no evidence of *Trichinella* spp.

The parasites observed in the boars assessed in this study show similarities and differences with those identified in other wild boar studies in other countries. A study of wild boar infections with *Strongylus* or *A. suum* in Finland was reported (Hälli et al., 2010). A study by Petersen et al., 2020 investigated Danish wild boars for pathogens transmissible to humans or domestic pigs (Petersen et al., 2020). No samples tested positive for *Brucella suis*, MRSA, *Salmonella*, or *Trichinella*. However, the boars had a high prevalence of various parasites, particularly *Eimeria* species and lungworms (*Metastrongylus* sp.), indicating a low risk for the former pathogens but a significant presence of parasites that could affect domestic pigs. Another study of wild boars in Russia's Primorsky Krai by Belov et al. (2022) found six parasite genera in their feces (Belov et al., 2022). The most common were the nematodes *Metastrongylus* spp. (13.6%) and *Trichuris suis* (7.6%), while other parasites like *A. suum* and *Eimeria* sp. were present at lower rates. No link was found between parasite prevalence and the age or sex of the boars.

In a recent similar study, Pilarczyk et al., 2024 compared gastrointestinal parasites in wild boars from urban and suburban areas in Poland (Pilarczyk et al., 2024). Both groups were infected with the same species of coccidia (*Eimeria*, *Isospora*) and nematodes (*Ascaris suum*, *Oesophagostomum dentatum*). Urban boars had a significantly higher prevalence of *Eimeria* (47.5% vs. 18.8%, *p* = 0.04) but a lower prevalence of nematodes than suburban boars. The high level of coccidia suggests a health threat to farm animals, while the presence of *A. suum* indicates a potential, though low, zoonotic risk to humans.

A previous study by Imrich et al. (2016) found a high prevalence of parasitic infections in wild boars in the Pohronský Inovec Mountains (Imrich et al., 2016). The prevalence of different parasite types in wild boars was as follows: *Metastrongylus* spp. 10%, *Hyostrongylus rubidus* 10%, *T. suis* 40%, *Physocephalus* spp. 10%, *A. suum* 50%, *S. ransomi* 10%, and *Eimeria* spp. 90%. However, the infection intensity was generally low for most parasites, except for *Eimeria*, which showed a higher oocyst count. Younger boars (under 30 kg) were found to be the most frequently infected individuals.

Furthermore, Senlik et al., 2011 investigated helminth infections in wild boars from Turkey, finding a high infection rate of 74% (Senlik et al., 2011). Twelve species of helminths were detected, with the following prevalence rates: *M. apri* (59%), *M. salmi* (52%), *M. pudendotectus* (52%), *D. dendriticum* (33%), *G. urosubulatus* (22%), *M. hirudinaceus* (19%), *G. pulchrum* (11%), *P. sexalatus* (7%), *T. suis* (7%), *A. strongylina* (4%), *H. rubidus* (4%), and *T. hydatigena* larvae (4%). Similar to our study, all tested muscle samples were negative for *Trichinella* larvae.

Moreover, Tamara et al., 2021 assessed parasitic infections in wild boars from two different hunting grounds in Serbia (Tamara et al., 2021). The presence of protozoa, *Eimeria* spp. *Isospora* spp. (76.38%; 32.26%) and *Balantidium coli* (7.08%), nematodes, *A. suum* (29.03%), *T. suis* (31.49%; 19.35%), *H. rubidus*, *Oesophagostomum* spp. (55.12%; 48.39%), *M. pudendotectus* (66.14%; 21.51%), *G. hispidum* (3.94%; 2.15%), and *M. hirudinaceus* (9.45%; 7.53%) and trematodes, *F. hepatica* (5.51%; 4.30%) and *D. dendriticum* (0.78%; 2.30%) were detected as single or mixed infections, via qualitative coprological examination. The study concluded that monitoring these infections is crucial to protecting both the hunting economy and public health, given the associated zoonotic risks.

A previous study of wild boars in Bulgaria identified a diverse parasite fauna, including ten helminth genera and the protozoan *Eimeria* (Panayotova-Pencheva and Dakova, 2018). Ten helminth genera (*Metastrongylus*, *Strongyloides*, *Oesophagostomum*, *Hyostrongylus*, *Globocephalus*, *Nematodirus*, *Ascaris*, *Ascarops*, *Trichuris, Macracanthorhynchus*) and one protozoan (*Eimeria*) were established through coproscopical investigations. Helminths of the species *M. elongatus*, *M. pudendotectus*, *M. salmi*, *G. urosubulatus*, *O. dentatum*, *O. quadrispinulatum*, *T. suis, M. hirudinaceus*, *A. suum*, *A. strongylina,* and *P. sexalatus* were found during the necropsies. *Metastrongylus*, *Globocephalus,* and *Oesophagostomum* were the genera with the highest infection prevalence, at 28.75%, 13.75%, and 12.5%, respectively. The research reports the first finding of *O. quadrispinulatum* in Bulgarian wild boars.

The differences in parasites in our study and other studies could be affected by diverse factors such as environmental conditions (season, temperature, humidity, precipitation, etc.), the presence or the lack of intermediate hosts for certain parasites, population density and dispersion, the number of wild boars within a region, and the number sampled in each study. While *Trichinella* was not seen, other zoonotic parasites were seen. Further studies are needed to understand the potential risks to human health posed by these infections.

While the present study identified several parasite species with known zoonotic potential (e.g., *F. hepatica*, *G. pulchrum*, and *Sarcocystis* spp.), the detection of a pathogen in a wild reservoir does not, by itself, constitute evidence of active transmission risk to humans or domestic animals in this region. The observed prevalence and intensity data must be interpreted within the local ecological context, including the boars' foraging behavior, habitat overlap with livestock, and the presence of intermediate hosts. For instance, the high prevalence of *M. hirudinaceus* (86.6%) and metastrongyles (over 50%) reflects dietary and environmental exposure rather than a direct public health threat, as these parasites require specific intermediate hosts and have limited spillover potential to humans under normal conditions. Similarly, the presence of *T. hydatigena* larvae indicates the boars' role as intermediate hosts in a sylvatic cycle with canids, but this finding is more relevant to wildlife management and domestic dog deworming programs than to human taeniasis. Therefore, rather than overemphasizing zoonotic risk, our findings underscore the importance of understanding wild boar parasitofauna as a bioindicator of ecosystem health, foraging ecology, and co-infection dynamics, factors that are often more informative for disease surveillance than crude prevalence lists. Future studies should incorporate molecular characterization, seasonal sampling, and host-density data to disentangle the complex interplay between wildlife, domestic animals, and environmental drivers of parasite transmission.

## 5 Conclusion

In conclusion, based on this study's findings, wild boar populations in Mazandaran province exhibit a high prevalence of internal parasitic infections, indicating active parasitic cycles in the region's natural habitats. These results introduce boars as an important and potential reservoir for parasitic diseases and emphasize the need for serious attention to the health management of these populations, as well as the implementation of integrated surveillance programs to assess the risk of transmission to domestic animals and humans in this strategic region of the country. This study provides a baseline for monitoring changes in parasite biodiversity and abundance, which may serve as early warning signals for ecological disturbances or shifts in wild boar–livestock interfaces. We recommend that future research adopt a longitudinal, multi-species approach that integrates parasitological, molecular, and spatial data to clarify transmission pathways and inform evidence-based management strategies that balance wildlife conservation, animal health, and human well-being without overstating zoonotic risks.

## CRediT authorship contribution statement

**Moein Abolhasani Drounkola:** Writing – review & editing, Writing – original draft, Visualization, Validation, Resources, Methodology, Investigation, Formal analysis, Data curation, Conceptualization. **Mohammad Reza Salimi Bejestani:** Writing – review & editing, Writing – original draft, Visualization, Validation, Supervision, Software, Resources, Project administration, Methodology, Investigation, Funding acquisition, Formal analysis, Data curation, Conceptualization. **Emad Changizi:** Visualization, Validation, Methodology, Investigation, Data curation.

## Consent to participate

Not applicable.

## Consent for publication

Not applicable.

## Ethical approval

All applicable international, national, and/or institutional guidelines for the care and use of animals were followed. CODE: IR.SU.REC.1404.23.

## Funding

No funding was received.

## Declaration of competing interest

The authors declare that they have no known competing financial interests or personal relationships that could have appeared to influence the work reported in this paper.

## Data Availability

The datasets generated during and/or analyzed during the current study are available from the corresponding author upon reasonable request.
